# Computationally scalable regression modeling for ultrahigh-dimensional omics data with ParProx

**DOI:** 10.1093/bib/bbab256

**Published:** 2021-07-13

**Authors:** Seyoon Ko, Ginny X Li, Hyungwon Choi, Joong-Ho Won

**Affiliations:** Department of Statistics, Seoul National University, Republic of Korea; Department of Medicine, National University of Singapore, Singapore; Department of Medicine, National University of Singapore, Singapore; Department of Statistics, Seoul National University, Republic of Korea

**Keywords:** ultrahigh-dimensional omics data, parallel computing, sparse regression, latent group lasso, proximal gradient

## Abstract

Statistical analysis of ultrahigh-dimensional omics scale data has long depended on univariate hypothesis testing. With growing data features and samples, the obvious next step is to establish multivariable association analysis as a routine method to describe genotype–phenotype association. Here we present ParProx, a state-of-the-art implementation to optimize overlapping and non-overlapping group lasso regression models for time-to-event and classification analysis, with selection of variables grouped by biological priors. ParProx enables multivariable model fitting for ultrahigh-dimensional data within an architecture for parallel or distributed computing via latent variable group representation. It thereby aims to produce interpretable regression models consistent with known biological relationships among independent variables, a property often explored *post hoc*, not during model estimation. Simulation studies clearly demonstrate the scalability of ParProx with graphics processing units in comparison to existing implementations. We illustrate the tool using three different omics data sets featuring moderate to large numbers of variables, where we use genomic regions and biological pathways as variable groups, rendering the selected independent variables directly interpretable with respect to those groups. ParProx is applicable to a wide range of studies using ultrahigh-dimensional omics data, from genome-wide association analysis to multi-omics studies where model estimation is computationally intractable with existing implementation.

## Introduction

Omics technologies are principal modalities in today’s systems biology and molecular research. However, since the arrival of microarray-based gene expression profiling techniques, clinical omics data sets have been modestly sized in a majority of biomedical studies. The high dimensionality of data relative to the small number of samples implied insufficient statistical information for feature space exploration by the multivariable statistical models or machine learning methods, undermining their utility in the association analysis or diagnosis and prognosis of diseases. With increasing throughput and decreasing cost of experimental platforms, however, the landscape is quickly transforming from the era of ‘small *n*, large *p*’ problems to a new era of ‘large *n*, very large *p*’ problems now. The arrival of the new era is perhaps best signaled by genome-wide association studies with genotypes at millions of loci and with a sample size greater than hundreds of thousands [1], or multi-omics studies with tens of thousands of tumor biopsies in the Cancer Genome Atlas (TCGA) [2]. The emergence of large-sample, high-dimensional studies provides opportunities for multivariable regression modeling to be used in general practice of omics data analysis.

There are two major challenges to be addressed in this path forward. First, modeling approaches need to incorporate interpretability of models as a major criterion for success. This is a practically important point as modern omics technologies provide data at increasingly high resolutions. Various omics platforms now report multiple variables that collectively represent an independent physical, chemical, or biological entity, such as multiple loci of sequence variants under a linkage disequilibrium block of a genes, various mRNA transcripts of a gene, or CpG islands in the regulatory regions of a gene. In most of these cases, multiple variables represent slightly different aspects of a molecule, and sometimes one data feature may belong to multiple groups. In searching for the best statistical model, it will be important to consider the grouping information in the estimation procedure, especially when a large number of competing models may attain similarly optimal performance. Second, most existing software packages require in-memory storage of full data and computation on central processing units (CPU), which limits the scale of analyzable problems on standard computer hardware. The breaking point is yet to be widely recognized by those who routinely perform omics data analysis as the computer hardware has improved over time and univariate analyses are prevalent. Nonetheless, this emerging reality poses implementation challenges for future development of biostatistical and bioinformatics tools. In this context, we address the scalability of large-scale multivariable linear regression analysis of omics data in this paper, accounting for complex variable group structures in pursuit of optimal, interpretable models.

In high-dimensional data, regularization of linear regression model seeks a simpler model via sparsity-inducing penalization. Traditionally, scalability in penalized regression models has been tackled by screening variables that do not contribute to explaining the variance in responses and applying the fitting procedure only to the remaining variables via coordinate descent [3–5]. Coordinate descent updates a single regression coefficient at a time, hence the per-iteration computational cost is low in case of separable penalties like the plain least absolute shrinkage and selection operator (lasso) [6, 7]. The R package glmnet [8] has been widely used for this purpose. Recently developed snpnet package [9, 10] combines a large-scale screening rule and glmnet to analyze compressed SNP data. The Julia package MendelIHT.jl pursues the same goal with iterative hard thresholding [11]. For omics data analysis, more structured penalties reflecting known information on predefined genomic regions, pathways or gene ontologies (GO) are desirable as discussed above, and the variable groups may even be overlapping. The R package grpreg implements a block coordinate descent (BCD) method for non-overlapping group penalties, where each group corresponds to a variable block updated for each iteration [12]. Its extension, grpregOverlap, supports overlapping groups [13].

However, the scalability of these (block) coordinate descent-based methods is limited since they are inherently sequential: each coordinate update depends on the preceding coordinates. A sweep of the entire coordinates takes a long time if the number of variables is large. Even if a screening rule is employed in each iteration, the number of variables screened out is small when the penalization level is low. Since it is customary to select a model from the entire regularization path obtained by varying the level of penalization, per-iteration screening may not reduce the computational burden in such a case.

A viable alternative for the scalability bottleneck is to employ an easily parallelizable optimization algorithm and run it on a parallel/distributed computing environment. Ideally, if all the coordinates can be updated simultaneously, then a sweep over all coordinates takes the same time as a single coordinate update. Even if only a group can be updated simultaneously at a time, the benefit of parallelization is large if the average group size is reasonably big. Recent advances in big data sciences have made such algorithms available, and the suitable computing environments, e.g. graphics processing units (GPUs) and cloud computing, a commodity [14]. Evidence has shown that computing the entire regularization path in a parallelized fashion does not take much more time than it takes to solve a single penalized regression problem of the same size, without the help of a screening rule [15].

Motivated by these considerations, here we present a new software package for fitting regularized linear regression models on high-dimensional clinical omics data, embodying an efficient optimization strategy and capability for parallel/distributed computing options. The implementation, called ParProx, fits latent group lasso penalized regression models for survival analysis or sample classification. During model fitting, variables are regularized by non-overlapping or overlapping group penalties specified by the user and the variables in the same group are penalized jointly to reflect known group information. More importantly, we implemented ParProx in the high-performance programming language Julia to allow for parallel computation with GPUs or distributed computing over cloud environments (Amazon Web Services, Google Cloud Platform, Microsoft Azure, etc.) natively, which enable the modeling for ultrahigh-dimensional data sets.

We demonstrate regression modeling of clinical omics data using ParProx through three application studies. In the first application, we present GPU-based computing to fit a Cox regression model of somatic mutation counts for overall survival outcome in 9707 patients in TCGA [2, 16]. Here we create mutation counts of ~56 000 DNA sequence segments in the codons and regulatory regions as independent variables and define sets of the sequence segments belonging to individual genes or gene pairs that are interaction partners at the protein level as variable groups for penalization in the group lasso regression. In the second application, we obtain a gene expression-based logistic regression model for pathological complete response (pCR) to neoadjuvant chemotherapy for breast cancer as binary outcome, using ~12 000 mRNA-level measurements of genes as independent variables and membership of individual genes to pathways/GO terms as the *overlapping* variable groups for structured regularization [17–20]. In the last application, we present a Cox regression analysis of 377 liver cancer patients using DNA methylation status of CpG islands in and out of coding regions as covariates. Each methylation probe represents a CpG island on the genome, and the probes present in the genomic location near a gene form a variable group. As certain chromosomal regions are densely populated by multiple genes, some probes belong to two or more adjacent genes and thus create overlap in the variable groups, i.e. between adjacent genes. The methylation array platform used by TCGA contains as many as >865 000 probes originally, but we have trimmed this data to 289 509 probes for demonstration purposes. Each of these three data sets takes up to 4.3 gigabytes of memory even after trimming. Unless carefully managed, this size of data may cause serious issues in memory allocation during reading and modifying data entries, model estimation and cross-validation, especially when there are overlaps among the variable groups.

## Methods

### Proximal gradient descent for regularized logistic and Cox regression models

We first describe the computational workflow of ParProx in the typical binary classification setting. The goal of the regression modeling is to understand the influence of *p* covariates $X=\big({X}^1,\dots, {X}^p\big)$ on the probability $\Pr \big(Y=1|X\big)$ of a sample belonging to class 1, where the two classes are labeled 0 and 1. In logistic regression, if there are *n* samples, given the observed label ${y}_i$ and covariates ${x}_i=\big({x_i}^1,\dots, {x_i}^p\big)$ for each individual sample $i$, the likelihood of the observed data is modeled based on the assumption that the log odds of the class membership is a linear combination of covariates, yielding the likelihood of the linear combination coefficient $\beta =\big({\beta}_1,\dots, {\beta}_p\big)$:

$$\begin{align*}& Lik\left(\beta \right)={\prod}_{i=1}^n{\left[1/\big(1+\exp \left({\sum}_{j=1}^p{\beta}_j{x_j}^i\right)\right]}^{1-{y}_i} \\ & {\left[\exp \left({\sum}_{j=1}^p{\beta}_j{x_j}^i\right)/\big(1+\exp \left({\sum}_{j=1}^p{\beta}_j{x_j}^i\right)\right]}^{y_i}. \end{align*}$$

If the number of covariates *p* is large, as is the case in omics data, it is customary and reasonable to assume that only a few independent variables determine the response. This is promoted by multiplying a prior probability to the likelihood that causes all but a few coefficients among $\big({\beta}_1,\dots, {\beta}_p\big)$ to be zero, a process known as regularization. This prior typically takes the form of $\pi \big(\beta \big)\propto \exp\ \big(-\lambda \big\Vert \beta \big\Vert \big)$, where $\big\Vert \beta \big\Vert$is some norm of the coefficient vector $\beta$. By taking the logarithm, the model-fitting procedure amounts to an optimization problem of minimizing

(1) \begin{equation*} -L\ \left(\beta \right)+\lambda \left\Vert \beta \right\Vert, \end{equation*}

where $L\big(\beta \big)={\sum}_{i=1}^n\big[{y}_i{\sum}_{j=1}^p{\beta}_j{x_i}^j-\log \big(1+\exp \big({\sum}_{j=1}^p{\beta}_j{x_i}^j\big)\big)\big]$. The regularization parameter $\lambda$ is typically selected via cross validation [21].

In a typical survival analysis setting, the goal is to understand the influence of *p* covariates $X=\big({X}^1,\dots, {X}^p\big)$ to the survival probability $S\big(y\ |\ X\big)=\Pr \big(Y>y\ |\ X\big)$ that the survival time $Y$ of a subject is longer than time $y$. In the Cox proportional hazards model [22], given the $i$th subject, $i=1,\dots, n$, with covariates ${x}_i=\big({x_i}^1,\dots, {x_i}^p\big)$, whose time-to-death ${t}_i$ or right-censoring time ${c}_i$ is measured so that the observed survival time is ${y}_i=\min \big({t}_i,{c}_i\big)$, the survival probability is equivalently modeled through the hazard function $h\big({y}_i\ |\ {x}_i\big)=-S^{\prime}\big({y}_i\ |\ {x}_i\big)/S\big({y}_i\ |\ {x}_i\big)$, where *S′* is the derivative of the survival function *S*:

$$ h\left({y}_i\ |\ {x}_i\right)={h}_0\big({y}_i\ \left|\ \beta \right)\exp \left({\sum}_{j=1}^p{\beta}_j{x_i}^j\right). $$

Here ${h}_0\big(y\ |\ \beta \big)$ is the unspecified baseline hazard function. That is, the covariates affect the hazard multiplicatively in such a way that a linear combination of covariates determines the strength of the multiplication. The coefficient vector of the linear combination is denoted by $\beta =\big({\beta}_1,\dots, {\beta}_p\big)$. Cox then proposes to get rid of the unknown baseline hazard in the fitting procedure by maximizing the partial likelihood

$$ PL\left(\beta \right)={\prod}_{i=1}^n{\left[\exp \left({\sum}_{j=1}^p{\beta}_j{x_i}^j\right)/{\sum}_{t:{y}_t>{y}_i}\exp \left({\sum}_{j=1}^p{\beta}_j{x_t}^j\right)\right]}^{\delta_i}, $$

where ${\delta}_i$ is the indicator that is 1 if ${t}_i\le{c}_i$, i.e. the event of sample *i* is observed, and 0 otherwise [22]. Like logistic regression, if the number of covariates $p$ is large, a prior of the form $\pi \big(\beta \big)\propto \exp \big(-\lambda \big\Vert \beta \big\Vert \big)$ is multiplied to the partial likelihood to promote a sparse model. The model fitting procedure then amounts to an optimization problem of minimizing

(2) \begin{equation*} -L\ \left(\beta \right)+\lambda \left\Vert \beta \right\Vert, \end{equation*}

where $L\big(\beta \big)={\sum}_{i=1}^n{\delta}_i\big[{\sum}_{j=1}^p{\beta}_j{x_i}^j-\log \big({\sum}_{t:{y}_t>{y}_i}\exp \big({\sum}_{j=1}^p{\beta}_j{x_t}^j\big)\big)\big]$, which takes the form of a generalized linear model, similar to the logistic regression problem (1). The regularization parameter $\lambda$ is typically selected via cross validation as problem (1).

If this objective function (1) or (2) of the optimization problem were differentiable in $\beta$, then the typical gradient descent method, which iteratively updates the current estimate of $\beta$ by moving slightly to the opposite direction of the vector of the first-order partial derivatives of the objective function, would eventually yield the correct estimate. Unfortunately, the objective functions (1) and (2) are not differentiable due to the norm $\big\Vert \beta \big\Vert$. Nevertheless, the term $L\big(\beta \big)$ in (1) and (2) is differentiable, hence an extension of the gradient descent method called the proximal gradient descent (PGD) can be applied instead. PGD for (1) or (2) consists of two steps [23]:

(i) Compute the gradient $\nabla L\big({\beta}^{(k)}\big)$ of $L\big(\beta \big)$ at the current estimate ${\beta}^{(k)}$ of the coefficient vector $\beta$.
(ii) Update the estimate by the formula

(3) \begin{equation*} {\beta}^{\left(k+1\right)}=\arg \underset{\beta }{\min}\left\{\lambda \left\Vert \beta \right\Vert +\frac{1}{2{\alpha}_k\ }\left\Vert \beta -\right({\beta}^{(k)}+{\alpha}_k\nabla L\left({\beta}^{(k)}\right){\left.\kern0em \right\Vert}_2^2\right\}, \end{equation*}

where $\big\Vert \cdotp{\big.\kern0em \big\Vert}_2^2$ in the right-hand side of equation (3) is squared Euclidean norm, i.e. ${\big\Vert x\big\Vert}_2^2={\big|{x}^1\big|}^2+\cdots +{\big|{x}^p\big|}^2$, and the argmin operator refers to the parameter value that minimizes the expression in the right-hand side. The scalar ${\alpha}_k$ is the step size that determines how far to move the estimate from the current candidate ${\beta}^{(k)}$. To be specific,

(4) \begin{align*}& \qquad \nabla L\left(\beta \right)={X}^T\left(y-p\right) \nonumber \\ & ={\sum}_{i=1}^n\left({y}_i-{p}_i\right){x}_i\ \mathrm{where}\ {p}_i=1/\left(1+\exp \left(-{\beta}^T{x}_i\right)\right) \end{align*}

for the logistic regression model, and

(5) \begin{equation*} \nabla L\left(\beta \right)={X}^T\left(I-P\right)\delta ={\sum}_{i=1}^n\left({\delta}_i-{\sum}_{k=1}^n{\pi}_{ik}{\delta}_k\right){x}_i, \end{equation*}

where $\delta ={\big({\delta}_1,\dots, {\delta}_n\big)}^T$, $P=\big({\pi}_{ij}\big)$ with ${\pi}_{ij}={I}_{\big\{{y}_i\ge{y}_j\big\}}{w}_i/\sum_{l:{y}_l\ge{y}_j}{w}_l$, and ${w}_i=\exp \big({\beta}^T{x}_i\big)$, for the Cox proportional hazards model.

The idea of PGD is to approximate $L\big(\beta \big)$ by a spherically shaped quadratic function tangential to $L\big(\beta \big)$ at ${\beta}^{(k)}$ and above it for all other values of $\beta$ and then minimize the approximate objective function. By iteratively doing so, the minimum of the original function (1) or (2) can be found (Supplementary Figure 1A available online at http://bib.oxfordjournals.org/) even when the objective function is not differentiable at the optimal solution. For many choices of the norm $\big\Vert \beta \big\Vert$, the right-hand side of the second step (3) takes a closed form expression despite its non-differentiability (This includes the latent group lasso penalty chosen for this paper. See below for detailed derivation.) Thus, the whole iterative procedure is almost as simple as the usual gradient descent method.

The convergence of PGD update (3) depends on the Lipschitz constant of the gradient $\nabla L$ of $L$. Both the logistic and proportional hazards models admit a negative log-(partial) likelihood $L\big(\beta \big)$ of the regression coefficient vector $\beta =\big({\beta}_1,{\beta}_2,\dots, {\beta}_p\big)\in{\mathbb{R}}^p$ that is differentiable and convex in $\beta$, where ${\mathbb{R}}^p$ means the set of *p* real numbers [26]. Furthermore, the gradient $\nabla L$ satisfies the Lipschitz condition ${\big\Vert \nabla L\big(\beta \big)-\nabla L\big({\beta}^{\prime}\big)\big\Vert}_2\le M{\big\Vert \beta -{\beta}^{\prime}\big\Vert}_2$ for some positive constant $M$, which can be chosen as an upper bound of $\big\Vert{\nabla}^2L\big(\beta \big){\big.\kern0em \big\Vert}_2$, the maximum singular value of the Hessian of $L$. For logistic regression, $\big\Vert{\nabla}^2L\big(\beta \big){\big.\kern0em \big\Vert}_2\le \frac{1}{4}{\big\Vert{X}^TX\big\Vert}_2$; for Cox’s proportional hazards model, $\big\Vert{\nabla}^2L\big(\beta \big){\big.\kern0em \big\Vert}_2\le 2{\big\Vert{X}^TX\big\Vert}_2$, where $X=\big({x}_i^j\big)$is the $n\times p$ data matrix [14]. Convergence of update (3) is guaranteed if ${\alpha}_k\in \big[\varepsilon, \frac{2}{M}-\varepsilon \big]$, where $\varepsilon \in \big(0,\min \big\{1,\frac{1}{M}\big\}\big)$ [23].

### Optimization with latent group lasso penalty

In order to encode the prior knowledge, we employ the latent group lasso penalty defined as follows [24]. Assume a collection $G$ of groups of genes is given. That is, $g\in G$ is a subset of all the gene indexes $\big\{1,2,\dots, p\big\}$. Let $\mid g\mid$ be the number of elements in $g$. Define a $\mid g\mid$-dimensional vector ${\gamma}_g$ (denoted by ${\gamma}_g\in{\mathbb{R}}^{\mid g\mid }$) and the linear map ${P}_g$ that maps ${\gamma}_g$ to a $p$-dimensional vector ${\beta}_g\in{\mathbb{R}}^p$ in such a way that the elements with indexes in $g$ are equal to those of ${\gamma}_g$ and all the other elements are zero. For example, if $g=\big\{2,3\big\}$ and ${\gamma}_g=\big(-1,1\big)$, then ${\beta}_g=\big(0,-1,1,0,\dots 0\big)$ and ${P}_g$ is a $p\times \mid g\mid$ matrix with 1 as the 2nd and 3rd diagonal components and all the other components being zero. Then, the desired penalty is

(6) \begin{align*} P\left(\beta \right)=\operatorname{inf}\left\{{\sum}_{g\in G}{\lambda}_g\left\Vert{\gamma}_g\right\Vert :{\gamma}_g\in{\mathbb{R}}^{\left|g\right|},{\sum}_{g\in G}{P}_g{\gamma}_g=\beta \right\}, {\lambda}_g>0. \end{align*}

In other words, the regression coefficient $\beta$ is decomposed as a sum of latent components ${\beta}_g={P}_g{\gamma}_g$ and it is the norm of these components that is penalized (note ${\big\Vert{\gamma}_g\big\Vert}_2={\big\Vert{\beta}_g\big\Vert}_2$). In this way, overlaps between the groups are allowed. When there is no overlap, penalty (6) reduces to the classical group lasso penalty [25]. Although the latter penalty may be straightforwardly defined for overlapping groups, it tends to select the complement of a union of groups—if two groups share a gene but one group is not selected, then the coefficient for the shared gene must be zero and the other group is only partially selected. The penalty (6), on the other hand, promotes the opposite and this property is desired for group selection.

We formulate the estimation problem for the logistic and Cox models under the penalty (6). It can be shown that $P\big(\beta \big)$ is indeed a norm [24]. Typically, the ${\lambda}_g$’s in (6) are set to be proportional to the group size and the $\lambda$ in (1) or (2), e.g. ${\lambda}_g=\lambda \surd \mid g\mid$. Hence the problem takes the form of minimizing (1) or (2). Since the penalty (6) also has a minimization form, the estimation problem can be formulated as the following optimization problem.

$$\begin{align*} & \underset{\beta, \gamma }{\min }-L\big(\beta \big)+\lambda{\sum}_{g\in G}\surd \big|g\big|{\big\Vert{\gamma}_g\big\Vert}_2 \quad \textrm{subject to}\\ & \quad \beta ={\sum}_{g\in G}{P}_g{\gamma}_{g}, \qquad \gamma ={\big({\gamma}_g\big)}_{g\in G}, \end{align*}$$

which can be equivalently written as an unconstrained optimization problem

(7) \begin{equation*} \underset{\gamma }{\min }-L\left( A\gamma \right)+\lambda{\sum}_{g\in G}\surd \left|g\right|{\left\Vert{\gamma}_g\right\Vert}_2, \end{equation*}

where $A={\big({P}_g\big)}_{g\in G}$ is the $p\times{\sum}_{g\in G}\mid g\mid$ 0–1 valued matrix such that $\beta = A\gamma ={\sum}_{g\in G}{P}_g{\gamma}_g$.

It can be shown that the second term $\lambda{\sum}_{g\in G}\surd \big|g\big|{\big\Vert{\gamma}_g\big\Vert}_2$ is a norm of the aggregated latent vector $\gamma ={\big({\gamma}_g\big)}_{g\in G}$ and hence problem (7) again has the same structure as problems (1) and (2), with $L$ replaced by $L\big(A\cdotp \big)$. Here we can apply the PGD algorithm to solve (7) efficiently. For iteration $k+1$, PGD updates ${\gamma}_g$ by the formula (3). Since there is no overlap between ${\gamma}_g$’s in $\gamma$, this update has a closed form:

$$\begin{align*} & \qquad{\gamma_g}^{\left(k+1\right)}=\arg \underset{\gamma_g}{\min} \\ & \left\{-{\left[\frac{\partial L\left(A{\gamma}^{(k)}\right)}{{\partial \gamma}_g}\right]}^T\left({\gamma}_g-{\gamma_g}^{(k)}\right)+\frac{1}{2{\alpha}_k}{\left\Vert{\gamma}_g-{\gamma}_g^{(k)}\right\Vert}_2^2+\lambda \sqrt{\left|g\right|{\left\Vert{\gamma}_g\right\Vert}_2}\right\} \\ & =\left(1\hbox{--} \frac{\alpha_k{\lambda}_g}{{\left\Vert{\gamma_g}^{(k)}-{\alpha}_k\frac{\partial L\left(A{\gamma}^{(k)}\right)}{\partial{\gamma}_g}\right\Vert}_2}\right)\left({\gamma_g}^{(k)}-{\alpha}_k\frac{\partial L\left(A{\gamma}^{(k)}\right)}{\partial{\gamma}_g}\right), \\ & \mathrm{if}\ {\left\Vert{\gamma_g}^{(k)}-{\alpha}_k\frac{\partial L\left(A{\gamma}^{(k)}\right)}{\partial{\gamma}_g}\right\Vert}_2>{\alpha}_k\lambda \surd \left|g\right|, \end{align*}$$

and

(8) \begin{equation*} {\gamma_g}^{\left(k+1\right)}=0,\qquad \qquad \qquad \mathrm{otherwise} \end{equation*}

for all $g\in G$. The derivative $\frac{\partial L\big(A{\gamma}^{(k)}\big)}{{\partial \gamma}_g}$ can be written as

$$ \frac{\partial L\left(A{\gamma}^{(k)}\right)}{{\partial \gamma}_g}={P}_g^T\nabla L\left( A\gamma \right). $$

where $\nabla L\big(\cdotp \big)$ is delineated in formulae (4) and (5) for logistic and Cox models, respectively.

### Parallel and distributed computation

From the point of view of computing, the combination of the latent group lasso penalty and PGD enables parallel computation (Supplementary Figure 1B available online at http://bib.oxfordjournals.org/), since both the gradient $\nabla L\big(\beta \big)$ and the closed form expression of (3) can be computed independently for each latent variable group ${\gamma}_g$. Furthermore, within a variable group, each component can also be updated in parallel. An important implication of this doubly parallel feature of our approach in omics analysis is that the data set does not need to reside on a single storage—it can be split and stored in a distributed fashion in multiple computing devices. Each device can report the update of the estimate of the regression coefficients in parallel to the master device holding the tally of the objective function, independent of the others. The size of the data to analyze scales linearly with the number of devices, with negligible sacrifice in computing time. This is in a stark contrast with the BCD method of [12, 13] for optimizing the objectives (1) and (2), which, instead of approximately solving (7) for the whole vector $\gamma ={\big({\gamma}_g\big)}_{g\in G}$, solves it for one group at a time, i.e. BCD solves

$$ \underset{\gamma_g}{\min }-L\left({P}_g{\gamma}_g+{\sum}_{g^{\prime}\ne g}{P}_{g^{\prime }}{\gamma}_{g^{\prime }}\right)+\lambda \surd \left|g\right|{\left\Vert{\gamma}_g\right\Vert}_2 $$

for different $g$ for each iteration, sweeping the whole coordinates in $\mid G\mid$ iterations. Thus, BCD is inherently sequential and requires the whole dataset to be stored in a single device.

### Advantage of Julia

Julia is a high-level programming language that provides a syntax close to interpreter languages such as R and Python but employs dynamic type inference. Combined with its just-in-time (JIT) compilation feature, Julia can execute code very efficiently. A significant advantage of this design is that Julia solves the ‘two-language problem.’ Even though the syntax of R or Python is easy to follow, for the tasks requiring efficient and fast computation such as fitting large-scale penalized regression models, it is almost necessary to write the core computation layer in a low-level language such as C and Fortran, which is difficult to learn. Furthermore, seamless coordination of the two languages is limited by the external language interface of the host language, i.e. R or Python, which typically outdates the advance in hardware and compiler technology. Software packages glmnet, snpnet and grpreg are no exception. In Julia, one can write code close to the speed of compiled languages while maintaining the easy-to-use syntax of interpreter languages.

Another helpful feature of Julia is multiple dispatch, which allows multiple implementations of a function with different types of arguments. Then, the actual implementation (‘method’) executed in run time is selected based on the combination of the argument types. Multiple dispatch allows a code with an easy-to-read syntax to be optimally compiled to run on various hardware with only minor, high-level, changes; its implication is that many types of parallelization and distributed computation (e.g. GPU or cloud) can be supported seamlessly. In ParProx, the amount of changes necessary for GPU parallelization of PGD from simple, sequential implementation is contained to the change of array types and moving data between CPU and GPU. Supplementary Figure 2, available online at http://bib.oxfordjournals.org/, shows how data are exchanged between CPU and GPU while the parallel computation is conducted within GPU.

### Simulation study

To demonstrate the merit of ParProx, we first conducted simulation studies. Simulated datasets were generated to have possibly overlapping group structure. Following the design of [26, 27], we set $p$ independent variables to have a total of $R$ groups comprised of $S$ adjacent variables, in which $T$ variables overlap. In other words, the $j$th group consists of the $\big(\big(S-T\big)\big(j-1\big)+1\big)$th variable through the $\big(\big(S-T\big)\big(j-1\big)+S\big)$th variable. This design yields $p=\big(R-1\big)\big(S-T\big)+S$. For example, if $R=100$, $S=100$, and $T=10$, the first group consists of variables 1 through 100, the second group consists of variables 91 through 190, and the final 100th group consists of variables 8911 through 9010. We then set the regression coefficients ${\beta}_j={\big(-1\big)}^j\exp \big(-\frac{j-1}{W}\big)$, for $j=1,\cdots, p$. The parameter $W$ controls the effective sparsity of the true coefficients; later coordinates have negligible effect sizes. The entries of the $n\times p$ data matrix $X$ were sampled from the standard normal distribution. Binary outcomes were generated according to the logistic model $P\big({y}_i=1\big)=1/\big(1+\exp \big(-{x}_i^T\beta \big)\big)$ for $i=1,\cdots, n$. Survival times were generated based on the probability $P\big({T}_i>t\big)=\exp \big(-t\big)$. Right censoring was simulated by generating another set of $n$ times to events. If the censoring time was less than the corresponding survival time, the former replaces the latter, and the observation is marked censored. As a result, about half of the sample were censored. Since the software packages available for fitting logistic/Cox proportional hazards regression models with latent group lasso penalty are limited, we compared ParProx with grpreg (with no overlap) and grpregOverlap (with overlaps). For comparison with grpreg, we used $n=500$ simulated samples with $S\in \big\{10,100\big\}$, $T=0$, and set $R$ to make *P* = 200 000, 400 000, 600 000, 800 000, 1 200 000, 1 600 000 and 2 000 000. The effective sparsity parameter $W$ was chosen 10, 100, and 1000. For comparison with grpregOverlap, we chose $T\in \big\{2,10\big\}$, with the other parameters remained the same. For each combination of simulation parameters, the solution path consisting of 100 values of $\lambda$ was computed.

### Applications

We next illustrate the use of ParProx through three representative example data sets below, with detailed protocol in Supplementary Information, available online at http://bib.oxfordjournals.org/. Across the case studies of varying size, sparsity and group complexity, we show that ParProx fits group-regularized regression models and produces easily interpretable models with or without complex group penalties with arbitrary degrees of overlap. Another important innovation of this implementation is that the model-fitting process can be parallelized through distributed or parallel computing environments, if necessary, in case exorbitantly large data need to be analyzed without any screening before or during the optimization such as the strong rule in coordinate descent algorithms.

### Survival analysis with TCGA pan-cancer somatic mutation data

**Figure 1:**
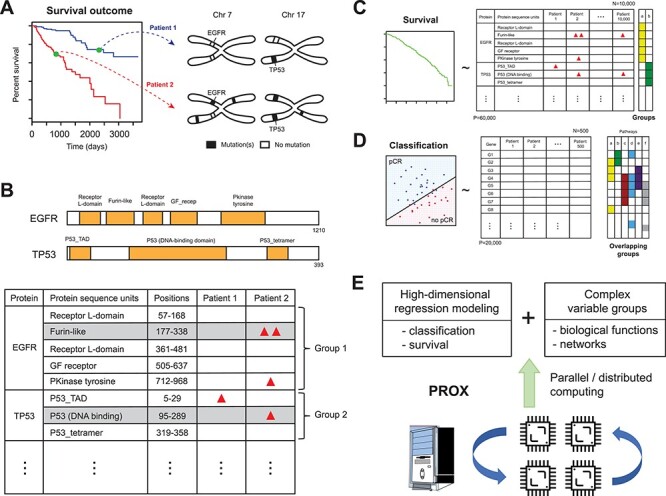
(**A**) Diagram of a hypothetical survival analysis with mutation data in EGFR and TP53 genes, two loci harboring somatic mutations with high frequencies in cancer genomes. Two people with different somatic mutation profiles may have two different cancer death risk. (**B**) The mutation counting method in the GPD framework. Mutations are counted by sequence regions encoding functional units of proteins such as protein domains. GPD mapping therefore produces sparse count data with inherent variable group structure, with genes serving as variables groups. (**C**, **D**) ParProx accommodates high-dimensional Cox regression as well as multi-group logistic regression for classification, with overlapping or non-overlapping group lasso penalties specified by the user. (**E**) ParProx fits overlapping group lasso regression models on large-scale data sets through distributed or parallel computing, if necessary, and handles overlapping variable groups through the proximal gradient optimization algorithm.

The first case study explores a multivariable Cox regression analysis using somatic mutation counts in protein coding regions as the predictors of cancer death risk. Since somatic mutations are sparse and not reproducibly detected at predetermined loci in early tumors, association between ‘locus-level’ somatic mutation data and clinical endpoints is not feasible. Alternatively, counts or rates of somatic mutations can be aggregated on predefined regions or individual genes [28, 29]. However, once mutations are aggregated *per* gene, it results in coarse interpretability in relation to the potential functional impact of mutations on the clinical outcome (Figure 1A). To find a reasonable compromise between the two options, we have recently proposed a functional region-based association testing approach for exome sequencing data, called gene-to-protein-to-disease (GPD) [30]. GPD counts mutations *per* genomic sequence segments pertaining to protein domains and 11 codons (33 bp)-long windows surrounding post-translational modification (PTM) sites and performs univariate statistical analyses with a clinical endpoint. Figure 1B illustrates how GPD summarizes mutation counts *per* protein sequence segments of three different types. These newly organized data can be used as covariates in the regression model.

Using this framework, we summarized the entire somatic mutation data into a count data set with 9707 patients and 55 961 protein sequence segments across the human exome. We then fitted a pan-cancer Cox regression model with 55 961 variables and group lasso penalties for overall survival of the 9707 patients using ParProx (Figure 1C). Here, all sequence segments in the same gene were set as variable groups, mirroring a hypothesis that individual mutations accrued in the same gene wield functional impact in different ways, but they are associated with the death risk of a given subject collectively. We also attempted the same regression analysis with group penalties imposed on groups of sequence segments present in pairs of genes which are proven to physically interact at the protein level.

#### Data preparation

TCGA pan-cancer somatic mutation data were downloaded from the Genomic Data Commons (MC3 Public MAF) [16]. In addition, we gathered 348 658 protein modification sites from PhosphoSitePlus [31] and 45 607 domains, families and repeats for 19 076 genes from Pfam [32]. Survival outcome data were downloaded from the TCGA Pan Cancer Clinical Data Resource [2], which contains curated clinical information for 10 793 patients. Among these, 367 non-primary skin cutaneous melanoma patients with metastatic tumors were excluded. Mapping somatic variants to protein units was described in our previous work [30]. Protein information units (PIU) refer to the genomic regions encoding protein domains, or ±5 amino acid-long windows around protein modification sites. Sequence regions between PIUs are defined as linker units (LU). The LUs include linker regions between domains as well as unannotated, repeat or disordered regions. The regions outside the protein-coding sequences including untranslated regions, introns and regulatory regions are collectively defined as noncoding units (NCU). NCUs are assigned to the closest gene in the genome. Aggregating somatic mutation mapped PIUs, LUs and NCUs from primary tumor samples categorized in 33 cancer types, we have 27 452 PIUs, 12 441 LUs and 16 068 NCUs, adding up to 55 961 units mapped by mutations from 9707 individuals.

#### Analysis of co-mutation frequency on protein interaction networks

The protein–protein interaction network has 133 146 unique pairs of interactions among 12 047 unique proteins. To estimate the significance of co-mutation frequency (the number of subjects having simultaneous mutations on both interacting proteins) of each pair of interacting proteins, we generated the null distribution of the frequency by randomly sampling 133 146 pairs of interaction from the pool of proteins 1000 times and calculating the co-mutation frequency for randomly sampled pairs in each iteration. The *P*-value of pair with co-mutation frequency *F* is defined as the number of pairs with co-mutation frequency higher than *F* divided by the total number of interactions (133 146), averaged across the 1000 iterations.

### Classification analysis with gene expression data in breast cancer therapy response

In the second example, we demonstrate that the overlapping latent group lasso model estimated by ParProx produces a biologically interpretable logistic regression model among many comparably predictive models, with the predictive signature consisting of genes associated with pCR to neoadjuvant chemotherapies, a binary outcome determined by expert pathologists (Figure 1D). In this conventional ‘small *n*, large *p*’ data example, we use biological pathways and GO terms as variable groups with arbitrary degree of overlap and nesting [33, 34], and show that the latent group lasso regression model optimized by ParProx identifies a sparse prognostic gene signature enriched with specific biological processes, rendering the prognostic model high interpretability over other similarly performing alternatives.

#### Data preparation

We downloaded gene expression microarray data sets with sample annotation information from the Gene Expression Omnibus database, based on the information from Prat *et al.* [20], Hatzis *et al*. from GSE25006 [17], Miyake *et al*. from GSE32646 [19] and Horak *et al*. from GSE 41998 [18]. Each data set was normalized by equalizing the median and median absolute deviation of expression values across the samples. For regression analysis, we applied logarithmic transform (base 2) and substracted the mean from expression values in each gene. For univariate differential expression analysis, we performed two-sample *t*-test and computed *q*-values [35] from the raw *P*-values to account for multiple testing.

#### Protein–protein interaction networks and gene pathways for variable group information

For variable group information in the TCGA somatic mutation data analysis with network penalty, we used protein–protein interaction network data from iRefIndex [36] and BioPlex [37]. For the group information used in the breast cancer data, we used a composite database of pathway databases called Consensus Pathway DataBase (CPDB) [34] and GO [33].

### Survival analysis with DNA methylation data in liver cancer

In the third example, we demonstrate ParProx in the context of ultrahigh-dimensional data, with *p* so large that parallel computing is required to fit a regression model with overlapping group penalties. We fit a Cox regression model with overlapping group lasso penalties on a DNA methylation data set from the liver hepatocellular carcinoma of TCGA. The DNA methylation array platform has probes representing genomic regions of high G/C content, and as such, the dimensionality is much higher than other omics data sets where the measurements are often summarized to individual gene level, e.g. gene expression or DNA copy number data. We show that ParProx can perform regularized Cox regression with penalties jointly applied to probes located in different segments of regulatory and coding regions for individual genes. In this data, we defined 90 099 groups over 289 509 variables (CpG islands). We show that the analysis can be completed within a reasonable amount of time using a single GPU, whereas another software grpregOverlap for overlapping latent group lasso regression analysis, implemented in R, could not handle the size of the data.

#### Data preparation

We downloaded the Illumina human methylation 450 array data set from Broad GDAC Firehose, corresponding to the liver hepatocellular carcinoma study (*N* = 428 from 377 unique patients) [38]. Fifty-two (52) patients had two biopsies and we used the primary tumor sample of those individuals for this illustration. We have selected 369 194 probes belonging to the following areas according to the manufacturer’s annotation: TSS1500, TSS200, 5′ UTR, 1st exon, gene body and 3′ UTR. Individual sequence regions were considered as variable groups (e.g. A1BG_TSS1500, A1BG_TSS200, A1BG_1st exon, A1BG_5′ UTR, A1BG_body, A1BG_3′ UTR are different variable groups). This specification resulted in a total of 90 099 groups, reflecting on average four methylation probes *per* group. In genomic regions with dense population of genes, the adjacent groups sometimes shared the same methylation probes, creating overlapping groups.

## Results

### Scalability of ParProx in simulation studies

**Figure 2:**
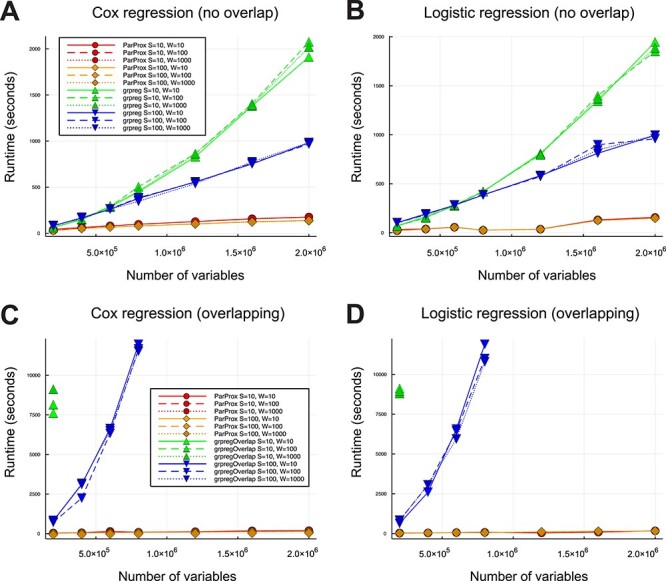
Time to convergence for the simulated runs of ParProx, grpreg and grpregOverlap showing the scalability of the former over the others. The parameters *S* and *W* control the size of variable groups and the effective sparsity of the true coefficients, respectively. Time was recorded in seconds. Lines in red and orange are for ParProx, and blue and green are for grpreg/grpregOverlap. (**A**) ParProx and grpreg with non-overlapping groups for Cox regression. (**B**) ParProx and grpreg with non-overlapping groups for logistic regression. (**C**) ParProx and grpregOverlap with overlapping groups for Cox regression. (**D**) ParProx and grpregOverlap with overlapping groups for logistic regression.

The running times until convergence of the algorithms for each combination of the simulation parameters are plotted in Figure 2. In general, ParProx ran on the Nvidia Titan V GPU was substantially faster than grpreg and grpregOverlap ran on the Intel Xeon Silver 4114 CPU. The combined benefit of parallelism and the PGD method gets larger with the number of variables $p.$ The performance gap is much larger when there are overlaps among the groups; grpregOverlap could not complete the fitting within a reasonable time span when the dimension is greater than a million. While for both grpreg and grpregOverlap, the running time clearly increases super-linearly with the dimension $p$, ParProx scales well with the dimension, sometimes sub-linearly. Note also that the effective level of sparsity did not affect the time until convergence significantly.

### Pan-cancer survival analysis of somatic mutations using group lasso Cox regression

#### Non-overlapping groups

We next demonstrate ParProx through survival analysis of somatic exome mutation data in TCGA pan-cancer cohort. As mentioned in the Applications section, we have mapped all somatic mutations curated by the Pan-Cancer consortium of TCGA to human protein sequence segments. These include (i) PIUs including 26 115 unique protein domains and 1337 segments surrounding PTM sites, (ii) unannotated regions called LUs, and non-coding regions (NCUs). Of 10 793 patients, 9707 tumors had at least one somatic mutation across 55 961 sequence segments. The 9707 × 55 961 count data matrix was used as the covariates in a Cox regression model of all-cause mortality, which requires 4.3 gigabytes of memory as double-precision floating-point numbers. We set 18 250 genes as variable groups for regularization in the present analysis, but this group structure can be extended with overlap within ParProx, such as multiple genes in a biological process, biochemical pathway, or protein complex as a group as we demonstrate later. In addition to the mutation counts, we have adjusted the model for age at diagnosis, gender, and cancer type without regularization on these coefficients, as they are known cancer death risk factors and the overall survival rates vary widely across different cancers.

The 10-fold cross-validation for selecting optimal regularization parameter took 57 min in this data, and the final fit with the optimal regularization parameter took 16 min on an Nvidia Titan V GPU. The GPU experiments were run on a workstation with two 2.20 GHz 10-core Intel Xeon Silver 4114 CPU with 192GB memory, with four Nvidia Titan V GPUs with 8GB memory each attached. A detailed manual for ParProx analysis of this data set, using CPU and GPU, can be found in the software manual provided as Supplementary Information, available online at http://bib.oxfordjournals.org/. A similar non-overlapping latent group lasso regression model could be fitted using the grpreg R package with CPU [12], and the analysis took 2.8 min for solution path calculation with 100 values of the regularization parameter $\lambda$ and 28 min for 10-fold cross-validation (iMac desktop with 3.7 GHz 6-core Intel Core i5 processor and 32 GB 2667 MHz DDR4 memory).

Although grpreg was faster than ParProx for this size of data with non-overlapping groups, likely due to the overhead of data transfer from CPU to GPU dominating the merit of parallel computation in ParProx in the present example, the selected model by the grpreg was counterintuitive in several aspects. First, the selected model included a very small number of variables (Supplementary Table 1 available online at http://bib.oxfordjournals.org/), with almost all coefficients of mutation harboring sequence segments being negative. Second, the coefficients for cancer type, which adjust for varying relative risk of death in different cancers, were all negative except leukemia (LAML), although there are other cancers that are just as lethal as the baseline cancer (GBM). Third, the selected model did not contain the most well-known cancer death-associated protein domains on *TP53* and *EGFR* genes. All put together, we suspect that this aberrant result has to do with the default data transformation step (orthonormalization; see Discussion), which may not be applicable for non-continuously scaled variables (count data with a large number of zeros). ParProx does not apply orthonormalization to the data.

**Figure 3:**
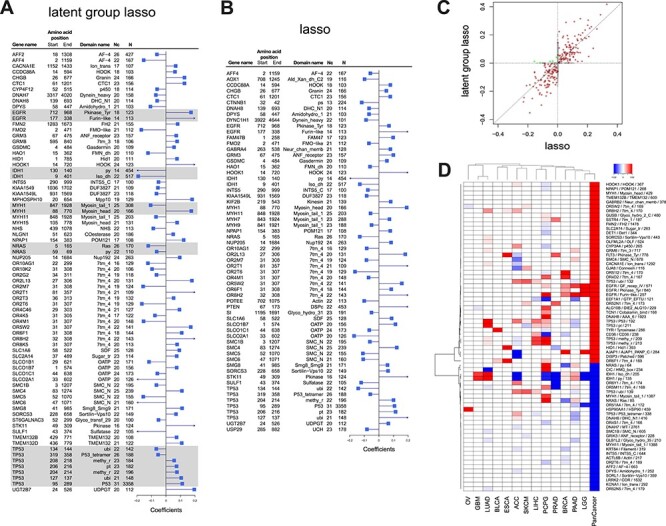
Cox regression coefficients from the model with (**A**) non-overlapping group lasso penalty (with proteins as groups) and (**B**) lasso penalty. Sequence segments from the same genes, jointly selected by regularization, are highlighted in gray boxes. The columns on the left-hand side of the barplot show gene identifiers, start and end position on respective protein sequences, protein domain or modification site information, the number of cancers with at least one patient with somatic mutations in the sequence segments, and the total number of patients with mutations in the segment, respectively. The segments are shown in the figure if mutations were detected in at least 10 cancers and regression coefficients are >0.05 in absolute value (**C**) Comparison of Cox regression coefficients between the two models. Brown and green dots show sequence segments with selected coefficients with consistent and inconsistent signs, respectively. Gray dots are the sequence segments selected in one of the two models only. (**D**) Comparison of Cox regression coefficients in the group lasso Cox regression in pan-cancer analysis as well as individual cancer analysis.

Figure 3 shows the covariates selected by group lasso regression of ParProx. The visualization in Figure 3A and B was confined to the selected variables representing protein domains or PTM sites, of absolute values greater than 0.01, and with mutations present in patients of at least 10 different cancer types (see Supplementary Table 1 for the full list available online at http://bib.oxfordjournals.org/). The group lasso regression selected 2370 variables (sequence segments) with non-zero coefficients (1131 PIUs, 492 LUs, 747 NCUs). Not surprisingly, the P53 domain on *TP53* gene, the most commonly mutated protein domain across 34.3% of all tumors (3358 tumors of 31 different types in the pan-cancer cohort), was determined to have a large deleterious effect on the cancer death risk, adjusting for other somatic mutation events across the genome. Two protein domains on *EGFR*, namely Furin-like domain and growth factor receptor IV domain on *EGFR*, also had comparably large positive coefficients (deleterious), although somatic mutations on these domains of *EGFR* were observed in specific cancers with much lower frequencies (14 of 33 types).

#### Overlapping groups

We next tested whether ParProx can handle a more complex group penalty structure with overlap. In the analysis above, the membership of sequence segments (PIU, LU, NCU and PTM site windows) to genes did not have any overlap in the group assignment. This time around, we gathered high confidence protein–protein physical interactions from two widely used databases (see Applications) and used the shared membership of sequence segments to any pair of two interacting proteins as a variable group. This analysis tests the hypothesis that co-mutations on two physically binding proteins in the same individual are likely to impact protein functions and thus the simultaneous mutation events have a greater deleterious or protective impact on cancer death risk. The mapping from our data translated into a total of 197 259 overlapping variable groups, many of them sharing the same sequence units. In other words, each sequence segment of a protein-coding gene may belong to two or more groups if the protein has multiple interaction partners.

Using a Nvidia Titan V, the analysis took 167 min in total with 10-fold cross-validation and final model fitting. A similar Cox latent group lasso analysis could not be performed by grpregOverlap R package [13], an extension to the grpreg package for handling overlapping latent group penalty, on the iMac desktop computer (‘vector memory exhausted’ error). Furthermore, the optimal regularization parameter selected by 10-fold cross-validation led to a Cox regression model with no sequence segments. In other words, we did not identify a signature of simultaneous mutations occurring on physically interacting proteins associated with survival, when adjusted for one another. This result can be interpreted in two different ways. It is possible that cancer death risk-associated somatic mutations do not necessarily co-localize to genes encoding members of the same protein complex, especially in the somatic mutation data of early primary tumors at the time of diagnosis. In fact, when we examined co-mutation events in patients with death within 5 years of follow-up, only 126 pair of interacting proteins had simultaneous mutations in more than 10 such patients. Further restricting to the patients who were deceased within 2 years, we had only 71 protein interactions with simultaneous mutations (Supplementary Table 2 available online at http://bib.oxfordjournals.org/).

Alternatively, it is also possible that tumors collected from primary diagnosis have a very low probability of harboring functionally consequential mutations on two or more essential members of a protein complex, and such events would not have been observed frequently in the early primary tumor collection of TCGA in the first place. Indeed, when we compared the number of interacting protein pairs with simultaneous mutations across the patients, we observed that only 8682 out of 133 146 total interaction pairs (6.5%) have more frequent simultaneous mutations than expected by random co-mutation on any pairs of proteins (more than 10 subjects). (Supplementary Table 2 available online at http://bib.oxfordjournals.org/ and Methods).

In either case, a key point here is that the variable group information for regularized regression makes a difference in the final model selection and overlapping latent group lasso allows users to specify different hypothesis in the model fitting based on appropriate biological priors. In this context, ParProx can handle the optimization problem that was not solved by a BCD-based implementation in R, the commonly used statistical analysis environment.

#### Benchmark: Plain lasso

To benchmark the non-overlapping group lasso model, we also ran the same regression with Cox lasso regression using ParProx, i.e. with *L*_1_ penalty on individual sequence segments but no group-wise regularization [39] (Supplementary Table 1 available online at http://bib.oxfordjournals.org/). As expected, the lasso model was essentially a sub-model of the group lasso model (Figure 3C, gray dots on the vertical and horizontal axes). The overlapping group lasso model selected 861 sequence segments as a prognostic signature of cancer death risk, whereas the lasso model selected 288 sequence segments. The deflation in the number of selected sequence segments was expected since group lasso would maintain a sequence segment as a predictor as long as there is one sequence segment of prognostic signal in the same protein.

However, among the sequence segments selected by lasso, the magnitude of coefficients for some variables belonging to the same gene was different between the two models. For example, the PTM sites (serine/threonine phosphorylation and lysine ubiquitination at respective sites) are all physically nested within the P53 domain, but lasso assigned the highest coefficient to the ubiquitination site (amino acid positions 127–138) rather than the P53 domain. Differences were also present in other genes such as the *EGFR* gene, where lasso regression assigned much greater coefficient to the Furin-like domain and deprecated the coefficient for the tyrosine kinase domain (Pkinase_Tyr) (Supplementary Table 1 available online at http://bib.oxfordjournals.org/). Across all other genes, the estimated coefficients seem to follow a consistent pattern: group lasso distributes the effect sizes more evenly to different members (variables) under the same selected group.

We next examined the overlapping group lasso regression coefficients from the pan-cancer analysis with those models fit on individual cancer data separately. Figure 3D shows the heatmap of regularized coefficients obtained from the pan-cancer analysis as well as those from analyses of individual cancer data. As expected, the sign of the coefficients was highly congruent among different analyses, although there were a few exceptions. Hence, we conclude that the pan-cancer survival analysis by the latent group lasso regression of ParProx successfully pools shared effects of mutations on the risk of cancer death in most, if not all, cases.

### pCR prediction analysis of gene expression data using overlapping group lasso logistic regression

**Figure 4:**
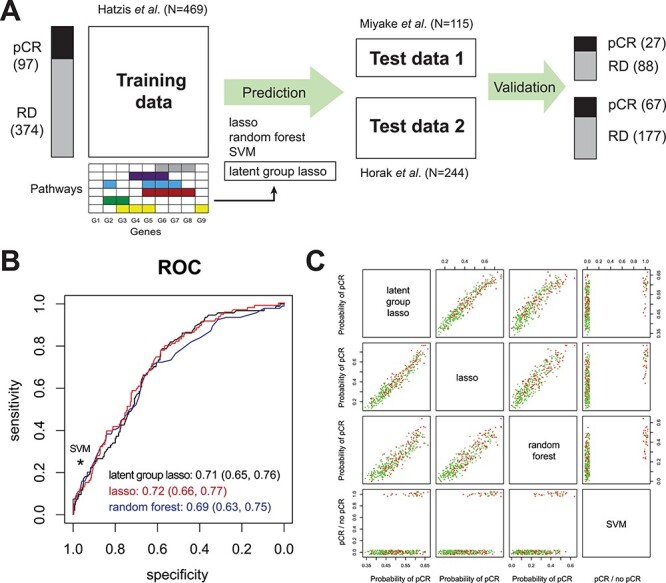
(**A**) Gene expression-based classification analysis of pCR in breast cancer. Lasso regression, RF, and SVM with radial basis function were used for benchmarking of pathway-based group lasso regression model. (**B**) Receiver operating characteristic curves of lasso, group lasso, and RF, and the sensitivity and specificity of SVM, all evaluated using the two test data sets. All four methods perform similarly in the classification of pCR and residual disease. (**C**) Class probabilities for the samples in the two test data sets reported by the four methods show highly similar results.

In the next case study, we demonstrate ParProx for acquiring a logistic regression classifier. Re-analyzing the meta-analysis data of Prat *et al*. [20], we aim to identify an mRNA gene expression signature to classify breast cancer patients undergoing chemotherapy with anthracycline and neoadjuvant agents into two groups, i.e. pCR and residual disease (RD). Here we use gene expression data sets of 12 307 genes and 469 patients in the training data set [17] and two test data sets [18, 19] (*N* = 115 and *N* = 244), and we use the pathways and GO terms as variable group information in the logistic group lasso regression. The analysis workflow is visually represented in Figure 4A.

Before fitting the regression model, we first carried out classical univariate analysis by gene-wise hypothesis testing in the training data by Hatzis *et al*. (*N* = 469) (see Applications for selection criteria). The analysis found as many as 1825 genes over-expressed and 1524 genes under-expressed in tumors from patients who achieved pCR compared to those with residual disease (*q*-value < 0.01, Supplementary Table 3 available online at http://bib.oxfordjournals.org/). The genes over-expressed in pCR patients showed enrichment of biological processes related to cell cycle, DNA repair, cell proliferation and protein folding, whereas the genes under-expressed in pCR patients showed enrichment for less essential pathways such as cilium assembly and extra cellular matrix (ECM) organization. This ‘routine’ analysis via hypothesis testing suggests that the tumors responding to the neoadjuvant agents with pCR have gene expression profiles favoring cell proliferation, while the tumors not achieving it do not.

We next built classifiers of pCR using four different methods: logistic regression with the plain lasso penalty, logistic regression with pathway-level overlapping latent group lasso penalty (ParProx and grpregOverlap), random forest (RF) [40] and support vector machine (SVM) [41]. In ParProx analysis, we used external data resource that combined multiple pathway databases to define variable groups, resulting in 11 734 groups among the 12 307 variables (including those singletons that do not belong to any pathway or GO term). With smaller size of the data set (12 307 by 469), the analysis was performed within a reasonable amount of time with ParProx on a GPU (12.5 min for cross-validation, 8.7 min for final model fitting). A similar analysis could be performed using the grpregOverlap package in R (28 min for cross-validation, 28 min for entire solution path calculation). As shown in Figure 4A, we trained the classifiers in the training data by Hatzis *et al*., and made prediction of pCR on the two test data sets. When we compared the area under the curve of the receiver operating characteristic (ROC), the first three methods performed as well as one another (Figure 4B), and the predictions from the SVM with radial basis kernel, with cost and gamma parameters optimized through 10-fold cross-validation within the training data, did not perform better than the three methods (scores shown in Figure 4C).

Given the highly similar performance metrics across different methods, we next investigated the interpretability of the gene expression signatures. Since the two machine learning methods with greater complexity (RF and SVM) utilize all features in the respective classifiers, we did not pursue interpretation of the underlying predictors, although it may be possible to prioritize variables, i.e. based on variance importance factors in the case of RF. Instead, we compared the selected genes between the two logistic regression models with and without group penalties. Logistic regression with the plain lasso penalty selected a total of 290 genes in the predictive signature (182 with positive and 108 negative coefficients, Supplementary Table 4 available online at http://bib.oxfordjournals.org/). Subsequent pathway enrichment analysis showed that the genes with positive regression coefficients, those contributing to the better chance of pCR, had mild enrichment of mitotic cell cycle and DNA replication genes, whereas the genes with negative coefficients were not particularly enriched in any known pathways other than ECM organization.

**Figure 5:**
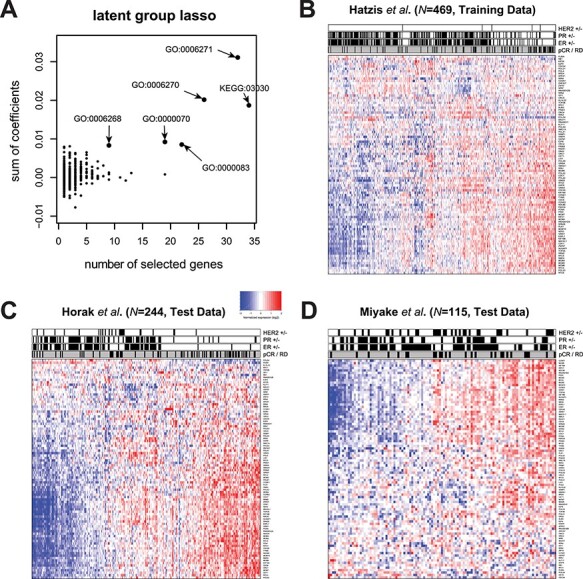
(**A**) Groups (pathways) containing at least one member gene selected with non-zero coefficients in the group lasso logistic regression. The GO terms with nine or more genes with sum of coefficients above 0.05, with positive contribution to the probability of pCR, are shown with arrows. (**B**–**D**) Heatmaps of the 90 member genes of the selected pathways in the training and test data sets. Each heatmap is annotated in terms of ER, PR and HER2 status, as well as pathologist-graded pCR status (outcome).

By contrast, ParProx analysis incorporating the pathway membership of genes selected a total of 830 genes (489 positive and 341 negative), a larger panel of genes than the lasso logistic regression classifier above. As stated in the previous case study, this is an expected consequence of using the group penalty, which tends to select genes in the same pathway together if there is a true effect of pathway-wide gene regulation. A clear advantage of the latent group lasso penalty is that one can rank pathways based on the number of genes with non-zero coefficients (Figure 5A). We selected five GO terms and one KEGG pathways with the largest number of genes with non-zero coefficients and large magnitudes in the sum of coefficients, with all six related to one overarching theme and sharing many common genes — DNA replication during mitotic cell cycle (Supplementary Table 4 available online at http://bib.oxfordjournals.org/).

Figure 5B shows the gene expression data with each gene normalized by its mean expression value, along with immunohistochemistry results of estrogen and progesterone receptors (ER and PR), fluorescence *in situ* hybridization analysis of HER2, and pCR status. In this training data, the tumors with pCR were mostly triple negative tumors (ER-, PR- and HER2-) as Prat *et al*. initially observed, indicating that the gene signature obtained by ParProx for a high chance of pCR is negatively associated with the positive hormone receptor status, hence positively associated with the canonical pattern of gene expression regulation for DNA replication and cell cycle progression in the triple negative tumors. Consistent with this, we observed that the gene signature in the test data set showed better concordance with the pCR status of patients in Horak *et al*. with at least half the patients classified under basal-like cancer with majority being triple negative (Figure 5C) than the status of patients in Miyake *et al*., where many were ER positive (Figure 5D).

In summary, this data example represents a case of logistic regression classifier with high-dimensional feature data with a modest sample size, with penalties imposed on a large number of overlapping variable groups. ParProx successfully optimized the objective functions under the constraint of overlapping, complex variable group information, with comparable computation time to an existing R package (grpregOverlap) which produced a much sparser predictive model with 22 covariates only (Supplementary Table 4 available online at http://bib.oxfordjournals.org/). In this data set, the classification performance was similar to the logistic regression with the plain lasso penalty, as well as other machine learning methods including RF and SVM in this data. Among these similarly informative models, however, the lasso logistic regression selected a gene signature devoid of enrichment of biological functions associated with the clinical endpoint (pCR), and the two machine learning algorithms did not yield interpretation of predictive data features as the regression models do. By contrast, the overlapping latent group lasso model estimated by ParProx yielded a gene signature indicating high probability of pCR in patients with DNA repair and cell proliferation genes over-expressed in their tumors.

### Survival analysis of ultrahigh-dimensional epigenetic data using overlapping group lasso Cox regression

In the third data set, we fitted a Cox regression model with overall survival as outcome variable and DNA methylation probes located in distinct genomic positions relative to protein coding genes as predictor variables in a liver cancer data. In this data set, even after selecting the probes located near protein coding genes only, the number of data features (*p*) was 289 508, with sample size of *N* = 377. In addition, we treated 90 099 genomic regions representing unique relative positions of probes as variable groups, including TSS1500, TSS200, 5′ UTR, 1st exon, gene body and 3′ UTR as annotated by the microarray vendor (see Applications). We thus guide the Cox regression model fitting to jointly penalize the probes in adjacent genomic regions.

Using overall survival as the clinical endpoint, we first attempted to fit overlapping group Cox regression using grpregOverlap in R. Unfortunately, the software was unable to perform model fitting and produced memory allocation errors in multiple desktop computers with at least 32GB RAM and 3.4 GHz quad-core intel i5 CPUs or better; see the ‘Comparison with existing software packages’ section. By contrast, ParProx was able to perform the *C*-index-based search of optimal λ value and the final model fit in 127 min with parallel computation using a Nvidia Titan V, demonstrating its scalability.

**Figure 6:**
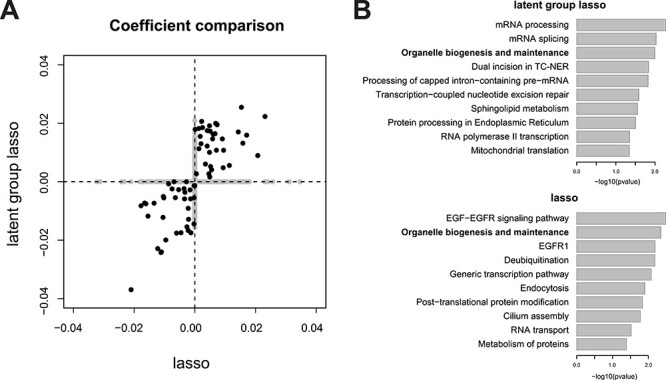
(**A**) Comparison of non-zero coefficients between group lasso and lasso models fitted using ParProx. Variables (CpG islands) with non-zero coefficients in one of the two models are shown in gray color along the horizontal and vertical axes. (**B**) Top 10 biological processes significantly enriched in the genes adjacent to the CpG islands selected by group lasso and lasso.

Par Prox reported an overlapping group lasso Cox model with 444 methylation probes located upstream and along the coding regions of 306 genes (see Figure 6A and Supplementary Table 5, available online at http://bib.oxfordjournals.org/, for data and regression coefficients, respectively). As in the previous two examples, the latent group lasso regression model produced an immediately interpretable model. The biological processes enriched in the genes close to the selected CpG island probes included response to stress, negative regulation of transcription from RNA polymerase II, apoptotic process, cell redox homeostasis and small molecule metabolic process. The model suggests that genes involved in oxidative stress response, metabolism and gene expression regulation are modulated by DNA methylation differently between patients with longer survival and those with shorter survival.

We next benchmarked the model against a Cox regression model with the plain lasso penalty, which selected 327 CpG island probes. To our surprise, the probes selected by Cox regression with lasso penalty had a poor overlap with the probes selected in the latent group lasso model, sharing only 70 common probes, albeit with good correlation (black circles in Figure 6A). In addition, *C*-index was comparable between the two models: 0.62(± 0.0) for both the latent group lasso penalty and the lasso penalty. Similar to the second application, this result likely suggests that there are a large number of weakly predictive regression models with different predictor variable combinations with comparable degrees of association with overall survival in the present data. Among those options, latent group lasso chose the model that best represents the variable group structure we specified as the modelers, and this prior resulted in a functionally different predictive model with respect to the genes associated with the epigenetic signatures (CpG islands) as shown in Figure 6B. This observation also reaffirms that specification of variable group structure influences the selection of data features associated with the clinical endpoint, and ParProx provides the interface to fit these models in ultrahigh-dimensional data sets that would otherwise have been impossible to fit.

## Discussion

In this work, we presented a scalable implementation to fit regression models for survival and classification analysis with structured group penalty representing biological prior information. The PGD method implemented in the Julia programming language can parallelize the iterative updates of the method in the case of large-scale data sets, which is the major advance offered by ParProx. We demonstrated the robustness of the implementation in both ‘large *n*, large *p*’ case (mutation data example) as well as ‘large *p*, small *n*’ case (gene expression data example) and showed that ParProx can deal with survival regression under the latent group lasso penalty using a very large-scale data set (*P* = 289 508) using parallel computing with GPU. Further, the simulation results indicate that ParProx is ready to embrace even larger size of data sets with millions of variables.

In contrast to the conventional differential expression analysis via hypothesis testing, our one-shot regression analysis strategy describes the multivariate relationship between clinical endpoint and high-dimensional molecular data using linear models. Linear models are often thought to be too restrictive to describe complex relationships between genotype and phenotype. However, it has the clear advantage of interpretability of results and low variance of prediction results. Linear models can summarize the overall impact of each variable onto the outcome into positive and negative values after accounting for the effect of others, and this directionality is often important for biological interpretation of predictive models. Despite the increasing popularity of machine learning and deep learning methods in omics data analysis, those methods permitting non-linear classifiers can only tell the importance of individual variables, but they fail to provide intuitive interpretation of the relationship between the outcome and the variables, as demonstrated in the breast cancer data as well. Within the class of linear models, ParProx provides an efficient solution to enable the challenging overlapping group lasso optimization and it has the appropriate software architecture for scaling to very large data sets.

Assessment of computation time between different implementations of linear models may be affected by the differences in algorithm, choice of grid coordinates for the regularization parameter, and convergence criteria, to name a few. Convergence criteria and grid selection are detailed below. Nonetheless, we emphasize that it is the choice of the algorithm that determines the scalability of the software. The proximal gradient method employed by ParProx is flexible in parallelization over distributed data, hence the computation time improves almost linearly with addition of hardware, e.g. GPU or a cluster node. This is the key contrast to grpreg and grpregOverlap packages implementing the BCD method, which is an inherently sequential algorithm. For even larger data than those studied in the paper, multiple GPUs or a virtual computer cluster on a cloud can be seamlessly employed in Julia, as demonstrated in Ko *et al.* [42]. We are planning to incorporate this feature in the next version of ParProx. In addition, when the sample size is large, stochastic approximation may be considered [43]. The gradient maps (4) and (5) both have the form of an average of per-sample gradients, i.e. $\nabla L\big(\beta \big)=\frac{1}{n}{\sum}_{i=1}^n\ell^{\prime}\big({x}_i^T\beta \big){x}_i$. Hence, each summand is an unbiased estimator of $\nabla L\big(\beta \big)$ and one may use this in place of the whole-sample gradient. Stochastic versions of PGD have been studied previously [44–46]. In the absence of the penalty, this method is well-known as the stochastic gradient descent (SGD). The downside of the sample scalability is that convergence is guaranteed only in a probabilistic sense, raising concerns on reproducibility, and slow.

We finally remark that the parallel PGD framework of ParProx can easily be extended to regression models other than logistic and Cox’s. In fact, most generalized linear models can be employed if the corresponding loss function has Lipschitz continuous gradient. For nonconvex penalties that can be expressed as a difference of convex functions, such as group minimax concave penalty (MCP) [47] and smoothly clipped absolute deviation (SCAD) [48], proximal averaging technique [49] can be incorporated into ParProx, at the expense of losing global optimality of the fit.

### Comparison with existing software packages

The popular software package glmnet for the R statistical computing environments fits linear, logistic and Cox regression models with the plain lasso penalty, and does not support penalties for arbitrary groups of variables. Its extension, snpnet, is specifically designed for large-scale SNP data compressed in PLINK2 format. The Julia software package MendelIHT.jl is also designed for compressed SNP data, and use the *L*_0_ penalty instead of the lasso. Variable groups are not supported.

The R software package grpreg fits linear, logistic and Cox regression models with non-overlapping group lasso penalties, hence solves problems (1) and (2). The package grpregOverlap extends grpreg to handle the latent group lasso penalty (6) to allow overlaps between groups, hence solves problem (7). The differences between these packages and ParProx are 3-fold: (a) solution algorithm, (b) memory management and (c) standardization of variables.

As for the algorithm, grpreg and grpregOverlap employ a BCD method instead of proximal gradient of ParProx. BCD is a simple algorithm that updates a (latent) variable group at a time with the other groups held fixed—each group update has a closed form. Hence, the complexity of each update is low. While inherently sequential, BCD is very efficient when the data size is moderate, as can be seen from the non-overlapping group-regularized analysis in the first case study.

However, overlapping groups, if they exist, may expand the data size considerably, causing memory issues even if the original data size is modest. For instance, in the first case study, the somatic mutation count data matrix is of size 9707 × 55 961. With a latent group penalty in which there are 197 259 overlapping groups, the number of latent variables becomes 1 384 850. grpregOverlap creates a new effective data matrix of size 9707 × 1 384 850 by duplicating the corresponding columns of the original data matrix in order to apply BCD, which requires more than 100 gigabytes of memory. On the other hand, ParProx evaluates the gradient of $L$ by using the original data matrix and the linear map $A$, which is sparse and only has 0/1 entries. Hence, the additional memory requirement is small. For a detailed comparison of memory requirements in ParProx and grpreg/grpregOverlap, see Table 1. Recall that it is the independent nature of update (8) that allows the use of GPU acceleration and other parallel and distributed computing environments, which is not feasible for BCD. Even if the original data matrix does not fit into the memory of a single device, it can be distributed over multiple devices and coefficients of each group over multiple devices, and the coefficients can be updated simultaneously.

**Table 1:** Minimum memory requirement for each case study and variable grouping method

	Predictive variables	Clinical outcome	Sample size	Variable size	Group overlap	Latent dimension	Data size	Minimum memory	
								grpreg/grpreg Overlap	ParProx
Case 1	Somatic mutation counts	Overall survival (Cox)	9707	55 963	No		4.4 GB	4.4 GB	4.4 GB
					Yes	1 384 805	4.4 GB	107.5 GB	4.4 GB
Case 2	Gene expression microarray	Pathological complete response (logisitic)	469	12 309	Yes	127 083	46.2 MB	476.8 MB	47.2 MB
Case 3	Methylation array probes	Overall survival (Cox)	377	289 508	Yes	370 473	991.3 MB	1.3 GB	994.2 MB

*Note*: The number of variables may differ from the numbers in the main text due to inclusion of additional risk factors (covariates) from outside the respective omics data, e.g. age and gender.

Finally, grpreg and grpregOverlap standardize variables by orthonormalizing the (latent) variables within the same group [3, 35], while ParProx employs the common practice of standardizing each observed variable. Through multiple example data sets, we have verified that the current implementation with orthonormalization seems to produce unexpected analysis results with or without overlapping groups when the variables are a mixture of continuous variables and non-continuous variables (categorial data and count data).

#### Convergence criteria

In all applications shown in the Results section, the PGD of ParProx was run until

$$ \frac{\left|f\left({\gamma}^{(k)}\right)-f\left({\gamma}^{\left(k-100\right)}\right)\right|}{\left|f\left({\gamma}^k\right)+1\right|}\le 5\times{10}^{-4} $$

for cross-validation, and run more stringently until

$$ \frac{\left|f\left({\gamma}^{(k)}\right)-f\left({\gamma}^{\left(k-100\right)}\right)\right|}{\left|f\left({\gamma}^k\right)+1\right|}\le 1\times{10}^{-5} $$

for fitting the final model after model selection. Here, $f\big(\gamma \big)$ denotes the objective function of the optimization problem (7). For the BCD method of grpreg/grpregOverlap, the default convergence criterion of the software was used, which stops the algorithm if

$$ \frac{{\left\Vert{\gamma}^{(k)}-{\gamma}^{\left(k-1\right)}\right\Vert}_2}{\surd p}\le 1\times{10}^{-4}. $$

#### Grid points for cross-validation

In ParProx, the regularization parameter $\lambda$ was chosen among 100 equally log-spaced $\lambda$ values between 10^−4^ and 10^−7^ in the first case study, among 100 equally log-spaced values between 10^–6.5^ and 10^–8.5^ in the second case study, and among 100 equally log-spaced values between 10^–5.5^ and 10^–7.5^ in the third case study. The grpreg and grpregOverlap packages automatically select 100 equally log-spaced values. The maximum is chosen to be the smallest $\lambda$ for which no variables are selected with the model. The minimum is 0.05 times the maximum value if there are more variables than the number of samples.

#### Option for excluding variables from regularization

Par Prox allows specification of variables to be excluded from regularization. This is a useful option in clinical omics data since certain variables represent known risk factors in a given disease context regardless of their statistical significance. Grpreg package allows the option of specifying variables excluded from regularization, but grpregOverlap does not offer it as of version 2.2-0.

**Key Points**

- Par Prox estimates non-overlapping and overlapping group lasso regression models as well as plain lasso regression models for survival and classification analysis of ultrahigh-dimensional omics data.
- Unlike existing implementations of the algorithms for fitting sparse regression models, ParProx embodies the proximal gradient method for optimization, which allows for parallelization over distributed data.
- Through specification of group lasso penalty, data analyst can impose prior information during variable selection, often resulting in in readily interpretable models.

## Supplementary Material

ParProx_Supplementary_Information_bbab256

SuppTable1_TCGApancancer_Cox_regression_bbab256

SuppTable2_PPIcoMutation_TCGAdata_bbab256

SuppTable3_BRCAneoadjuvant_univariate_analysis_bbab256

SuppTable4_BRCAneoadjuvant_logistic_regression_bbab256

SuppTable5_LiverCancer_DNAmethyl_Cox_regression_bbab256

## Software availability

Par Prox is freely available through GitHub repository at https://github.com/kose-y/ParProx.jl under the MIT license.

## Notes

**Seyoon Ko** was Ph.D. student in the Department of Statistics, Seoul National University, Republic of Korea at the time of writing. He is currently post-doctoral research fellow in the Department of Biostatistics, University of California, Los Angeles, USA.

**Ginny Xiaohe Li** was post-doctoral research fellow in the Department of Medicine, National University of Singapore, Singapore. She is currently post-doctoral fellow in the Department of Pathology, University of Michigan Medical School, Ann Arbor, MI, USA.

**Hyungwon Choi** is associate professor in the Department of Medicine, National University of Singapore, Singapore.

**Joong-Ho Won** is associate professor of statistics in the Department of Statistics, Seoul National University, Republic of Korea.
